# Clinical and epidemiological characteristics of cat scratch disease in children from southwestern China: a retrospective analysis of mNGS-confirmed cases

**DOI:** 10.3389/fpubh.2025.1743423

**Published:** 2026-01-20

**Authors:** Shu-yu Lai, Li Chang, Jia-xin Duan, Guang-lu Che, Qiu-xia Yang, Jie Teng, Hui Jian, Xiao-juan Liu, Fang Liu

**Affiliations:** 1 Department of Laboratory Medicine, West China Second University Hospital, Chengdu, China; 2 Key Laboratory of Birth Defects and Related Diseases of Women and Children (Sichuan University), Ministry of Education, Chengdu, China

**Keywords:** *Bartonella henselae*, cat scratch disease, metagenomic next-generation sequencing, pediatric infectious diseases, zoonoses

## Abstract

**Background and aim:**

Cat scratch disease (CSD) is a zoonotic infection predominantly caused by *Bartonella henselae*, typically featured by regional lymphadenopathy and febrile illness. Although these classic features characterize most cases, the clinical spectrum extends to severe systemic manifestations including meningitis and neuroretinitis, leading to poor prognosis. Given this potential for diverse clinical presentations, prompt microbiological confirmation becomes essential for accurate diagnosis and appropriate management of CSD. The present study aimed to provide a comprehensive analysis of epidemiological patterns, clinical characteristics, diagnostic findings, and therapeutic outcomes in pediatric CSD cases, with the ultimate goal of optimizing early detection and enhancing the clinical understanding of this disease.

**Methods:**

This single-center retrospective study analyzed 20 pediatric cases diagnosed with CSD at West China Second University Hospital in southwestern China between September 2021 and July 2025. All diagnoses were established based on comprehensive clinical evaluation including medical history, characteristic symptoms, and imaging findings. Definitive *B. henselae* identification was achieved through metagenomic next-generation sequencing (mNGS). These diagnostic characteristics were systematically evaluated and discussed in detail.

**Results:**

Among the 20 patients with CSD included in the study, 18 (90.00%) reported a history of cat contact. Ten patients were male (50.00%). School-aged children (6–14 years) accounted for the majority of patients. Eleven cases (55.00%) occurred in autumn. Fever and lymphadenopathy were the primary reasons for hospitalization. *B. henselae* was detected in all cases using mNGS. Nine patients were diagnosed with atypical CSD, seven of whom were female. Atypical CSD was associated with higher body temperature and longer hospitalization stay. Antimicrobial agents, including azithromycin, doxycycline, and rifampin, achieved satisfactory therapeutic outcomes.

**Conclusion:**

This study elucidates the epidemiological, clinical, and laboratory characteristics of CSD in children. mNGS may serve as a powerful tool to facilitate the diagnosis of CSD, including its atypical manifestations.

## Introduction

Cat scratch disease (CSD) is a zoonotic infection caused by *Bartonella henselae*, a gram-negative bacillus often found in cats (1). Cat-to-cat transmission of the organism occurs through cat flea, whereas cat-to-human transmission occurs primarily through contact with cats, including scratches or bites (2, 3).

The most common symptom of CSD is lymphadenopathy (4). CSD, generally considered a benign, self-limiting disease, usually presents with lymphadenopathy, fever, and fatigue. However, severe complications can occur and sometimes be life-threatening, especially in immunocompromised hosts (5, 6). Moreover, early and accurate diagnosis followed by prompt treatment is crucial given the nonspecific clinical manifestations of CSD, which can often mimic those of malignant conditions.

Granulomas and small abscesses detected on histological examination raise suspicion of CSD, but a definitive diagnosis requires microbiological verification (7). Traditional blood cultures have a sensitivity of only about 20% for isolating *Bartonella* species (8). The culture method is less sensitive and time-consuming and is therefore not routinely used for the diagnosis of CSD. Immunofluorescence and immunohistochemistry can serve as supplementary diagnostic tools, but the procedure is complex. Currently used laboratory methods for diagnosing CSD include the detection of specific antibodies in serum using indirect fluorescent antibody (IFA) and enzyme-linked immunosorbent assay (ELISA). However, these serological tests have low IgG sensitivity for *B. henselae* in patients with CSD (16–35%) and cannot distinguish current infection from previous infection. The sensitivity of immunoglobulin M (IgM) has increased (55–71.4%), but early detection may be negative due to the window period (9–12). *B, henselae* was detected using polymerase chain reaction (PCR) in some studies (13–15). However, the effectiveness of PCR relies on the presumption of the pathogen’s presence, which often depends on the patient’s accurate recollection of cat exposure history and the physician’s thorough understanding of CSD. Metagenomic next-generation sequencing (mNGS) is a comprehensive, unbiased method for detecting all pathogens in samples without prior selection. This technology has gradually been adopted in clinical practice. Our study included patients with suspected infections but atypical signs and symptoms; therefore, mNGS was employed for pathogen identification in all cases.

CSD is a globally recognized disease, studied in large-scale studies worldwide (16–19). A large number of case reports have been identified in China in recent years (20–22). Liu et al. summarized some Chinese CSD cases reported before 2012 (23). However, the economic conditions, public health infrastructure, lifestyle habits, and medical resources in China have changed significantly since then. These changes led to shifts in the clinical and epidemiological profiles of CSD in China. Our study aimed to systematically investigate the epidemiological characteristics, diagnostic approaches, and treatment strategies of CSD in 20 Chinese pediatric cases from 2021 to 2025. mNGS was adopted to assist in diagnosing all CSD cases.

## Materials and methods

### Patient enrollment

This single-center retrospective study was conducted at West China Second University Hospital, Sichuan University, a tertiary women and children’s specialty hospital in southwest China. The study cohort comprised the entire, consecutive series of pediatric patients (aged 0–14 years) identified from our hospital’s medical record system between September 2021 and July 2025 who met all of the following criteria: (1) a final diagnosis of CSD based on clinical manifestations and laboratory findings, (2) a positive *B, henselae* result confirmed by mNGS testing. No eligible patients were excluded. It was approved by the ethics committee of West China Second University Hospital, Sichuan University.

### Sample processing, library preparation, and sequencing

The samples were collected in accordance with standard aseptic procedures, processed immediately upon receipt at the laboratory or stored at −20 °C, and subjected to mNGS within 24 h. DNA was extracted using the QIAamp DNA Micro Kit (QIAGEN, Hilden, Germany) following the manufacturer’s protocols. DNA libraries were constructed using the QIAseq Ultralow Input Library Kit (Illumina, San Diego, CA, USA) following the manufacturer’s protocols. The quality of the library was assessed using a Qubit fluorometer (Thermo Fisher, Waltham, MA, USA) and an Agilent 2,100 Bioanalyzer (Agilent Technologies, Santa Clara, CA, USA). The high-quality genomic library was finally sequenced on the NextSeq 550 platform (Illumina).

### Statistical analysis

The statistical analysis of data was performed using SPSS Version 21.0 (IBM SPSS Corp., Armonk, NY, USA). Continuous variables with a normal distribution were expressed as the mean ± standard deviation and compared between groups using the t-test. Non-normally distributed continuous data were presented as median (interquartile range) and compared between groups using the Mann–Whitney *U* test. For categorical variables, the chi-square test or Fisher’s exact test was applied as appropriate. A *p* value less than 0.05 indicated a statistically significant difference.

## Results

### Patient characteristics

The cohort included 10 male and 10 female patients. Among these 20 patients with CSD, 18 (90.00%) reported a history of feline exposure. The age stratification of this cohort was as follows: <1 year (*n* = 0, 0%), 1–2 years (*n* = 1, 5.00%), 3–5 years (*n* = 4, 20.00%), and 6–14 years (*n* = 15, 75.00%). The predominance of school-aged children (6–14 years), who are more likely to have active feline contact, potentially elevates the risk of *B. henselae* infection (Table 1).

**Table 1:** Demographic and clinical characteristics of participants (*n* = 20).

Characteristics	Frequency, *n*	Percentage (%)
**Sex**		
Male	10/20	50.00
Female	10/20	50.00
**Age (year)**		
<1	0/20	0.00
1–2	1/20	5.00
3–5	4/20	20.00
6–14	15/20	75.00
**Cat/kitten contact history**		
Yes	18/20	90.00
Unknown	2/20	10.00
**Season of admission (month)**		
Spring (3–5)	3/20	15.00
Summer (6–8)	2/20	10.00
Autumn (9–11)	11/20	55.00
Winter (12–2)	4/20	20.00
**Cause of admission**		
Fever	15/20	75.00
Lymphadenopathy	5/20	25.00
**Febrile during the course**	17/20	85.00
**Peak temperature (°C)**		
≥40	12/17	70.59
<40	5/17	29.41
**Lymph node involvement**	11/20	55.00
Axillar	2/11	18.18
Cervical	3/11	27.27
Inguinal	1/11	9.09
Axillar + cervical	2/11	18.18
Axillar + supratrochlear	1/11	9.09
Cervical + supraclavicular	1/11	9.09
Axillar + cervical + inguinal	1/11	9.09
**Duration of symptoms at admission (day)**		
<7	1/20	5.00
7–14	9/20	45.00
15–30	10/20	50.00
**Length of hospital stay (day)**		
<7	2/20	10.00
7–14	13/20	65.00
15–30	5/20	25.00
**Treatment**		
Azithromycin	1/20	5.00
Azithromycin + Rifampicin	8/20	40.00
Doxycycline	1/20	5.00
Doxycycline + Rifampicin	10/20	50.00
**Outcome**		
Improved	20/20	100.00

### Seasonal and clinical presentation

The disease presentation followed a seasonal trend, with 11 cases (55.00%) occurring in autumn, suggesting a potential increase in disease risk in this season (Table 1).

Fifteen patients (75.00%) were admitted to the hospital due to recurrent fever lasting 8–23 days, whereas five (25.00%) presented with lymphadenopathy persisting for 6–30 days. All 20 patients had previously sought evaluation or treatment at local primary care facilities without satisfactory improvement, leading to their subsequent hospitalization. Following a comprehensive clinical evaluation, the number of febrile cases increased to 17 (85.00%), with 12 patients (70.59%) demonstrating high-grade fever (≥40 °C). Five patients (25.00%) were admitted to the hospital due to unexplained lymphadenopathy. After admission, lymphadenopathy was identified in 11 patients (55.00%) through physical examination and ultrasonography (Table 1).

### Laboratory findings

Of the 20 pediatric patients, 6 exhibited leukocytosis, 7 neutrophilia, 5 decreased hemoglobin levels, and 6 thrombocytosis, based on the reference intervals for blood cell analysis in children (Health Industry Standard of the People’s Republic of China, WS/T 779–2021). The results of the markers of systemic inflammation revealed an elevated C-reactive protein level (>8 mg/L) in 17 patients (85.00%), with a mean of 39.62 mg/L across all cases. All 16 tested cases had accelerated erythrocyte sedimentation rate. Procalcitonin levels were elevated in all five cases tested. Interferon-gamma release assays were negative in all 20 patients (Table 2).

**Table 2:** Laboratory findings.

Items	Cases above reference range (%)	Cases below reference range (%)
Blood routine test		
White blood cell count	6/20 (30.00)	0
Neutrophils count	7/20 (35.00)	0
Hemoglobin level	0/20 (0.00)	5/20 (25.00)
Platelet count	6/20 (30.00)	0
Inflammatory parameters		
CRP	17/20 (85.00)	/
ESR	16/16 (100.00)	/
PCT	5/5 (100.00)	/
IGRA positive	0/20 (0.00)	/
mNGS		
*Bartonella henselae* detected	20/20(100.00)	/

*B. henselae* was detected using mNGS in all specimens collected from the 20 patients. These included 16 blood samples, tissue biopsies from axillary, elbow, and cervical masses (one each), and one drainage fluid sample from an axillary mass. Additionally, herpesviruses, including Epstein–Barr virus (EBV) and cytomegalovirus (CMV), were identified with ≥ 5 unique reads in the blood samples of four patients (Table 2).

### Comparison of clinical characteristics between atypical and typical CSD cases

Nine pediatric cases presented with atypical manifestations beyond self-limited regional lymphadenopathy or lymphadenitis, including three cases of meningitis, one of meningitis and osteomyelitis, two of encephalitis, one of hepatosplenic involvement, one of retinitis and conjunctivitis, and one of retinal mass.

These patients with atypical CSD included more females (7/9, 77.78%) than males (2/9, 22.22%). Although this difference did not reach statistical significance in the current small-sample study (Fisher’s exact test, two-tailed, *p* = 0.070), this trend is worthy of verification in future larger-scale studies.

No significant difference in median age was found between patients with atypical CSD (9 years) and those with typical CSD (9 years) (Mann–Whitney *U* test, *p* = 0.261).

All patients with atypical CSD (9/9, 100%) developed fever during the course of illness, whereas 27.3% (3/11) of patients with typical CSD remained afebrile throughout the disease course. Furthermore, patients with atypical CSD had significantly higher maximum body temperatures (median = 40.40 °C) compared with those with typical CSD (median = 39.70 °C) (Mann–Whitney *U* test, *p* = 0.002).

Patients with atypical CSD had a significantly longer length of hospital stay (14.89 ± 5.44 days) compared to those with typical CSD (10.45 ± 3.91 days) (*t*-test, *p* = 0.048) (Table 3).

**Table 3:** Clinical characteristics between atypical and typical CSD cases.

Characteristics	Atypical CSD cases (%)	Typical CSD cases (%)	*P* value
Sex			0.070
Male	2 (22.22)	8 (72.72)	
Female	7 (77.78)	3 (27.27)	
Age (y), median (IQR)	9.00 (6.50–11.00)	9.00 (5.00–9.00)	0.261
Peak temperature (°C), median (IQR)	40.40 (40.15–40.60)	39.70 (36.60–40.00)	**0.002**
Length of hospital stay (day)	14.89 ± 5.44	10.45 ± 3.91	**0.048**

Bold letters indicate significant differences, CSD cat scratch disease, IQR interquartile range.

### Treatment and outcomes

All patients received antibiotic therapy. Two cases with typical CSD were treated with azithromycin or doxycycline monotherapy, whereas the remaining cases received combination therapy with azithromycin/doxycycline plus rifampin. All patients improved and were discharged from the hospital.

A total of 10 patients were followed up approximately 1 month after discharge. Among these, nine exhibited normal physical examination findings, complete blood count, CRP levels, and biochemical test results. One child with hepatic and renal involvement underwent unenhanced and contrast-enhanced abdominopelvic computed tomography on day 15 after discharge, which revealed an increase in the number and size of nodular enhancing lesions in the liver and spleen, as well as partial enlargement of para-aortic lymph nodes, compared with previous imaging. No subsequent medical records for this patient were accessible in the hospital information system.

## Discussion

This study enrolled 20 pediatric patients diagnosed with CSD at a tertiary children’s hospital in southwestern China between September 2021 and July 2025. Despite the small sample size, the study found that atypical CSD cases accounted for 45% and involved the central nervous system, bone marrow, eyes, liver, and spleen, providing rich clinical data.

A majority of the nine atypical CSD cases included in this study were female (7/9), consistent with the findings of Courtney et al. (16), who also reported a female predominance in patients with atypical CSD aged 0–14 years. Concurrent meningitis or encephalitis was observed in six cases, representing the most common complication, a finding consistent with the results of Reynolds et al. (24). Nevertheless, the limited sample size in this study necessitates further investigations with larger cohorts to uncover and investigate the atypical manifestations of CSD.

This study found that CSD mainly occurred in autumn. The reasons for this marked seasonality remain unclear, although human contact with flea-infested cats has been identified as a risk factor for CSD infection. As reported by Reynolds et al., cat fleas are prominent pests in summer and autumn across most of the continental United States (24). Zhou et al. (25) reported seasonal fluctuations in *Bartonella* prevalence among fleas in southeastern China, with the highest rates observed in October, followed by September.

*Bartonella* spp. are fastidious bacteria challenging to culture from clinical samples. Therefore, microbiological diagnosis typically relies on serological detection of anti-*Bartonella* antibodies or PCR-based detection of bacterial DNA (26). Currently, the definitive diagnosis of CSD largely depends on serological tests such as IFA and ELISA (2, 11, 27, 28). However, the reported sensitivity and specificity of these assays vary considerably. PCR is a molecular method with high sensitivity and specificity, demonstrating advantages in diagnosing CSD (29–32). Nevertheless, PCR requires presumptive selection of target pathogens, which can be difficult for clinicians, especially when dealing with atypical CSD presentations. In contrast, mNGS detects nucleic acids from all pathogens in a sample without prior targeting, potentially offering higher CSD detection rates than PCR. Currently, mNGS is more expensive. However, it has a shorter turnaround time, enables simultaneous detection of multiple pathogens, avoids the need for sequential pathogen-specific testing, and more effectively identifies co-infections, thereby facilitating timely and effective patient management. As reported by Habot-Wilner et al., the mNGS can comprehensively identify the pathogens of intraocular infections, thereby enhancing the accuracy of treatment strategies (33). The increasing clinical adoption of mNGS has enabled more CSD diagnoses to be confirmed using this technology (7, 34, 35).

All patients with CSD included in this study underwent mNGS. *B. henselae* was detected in either peripheral blood or biopsy/drainage specimens. Additionally, EBV was detected in three cases (with 5, 13, and 21 specific reads, respectively), and its presence was corroborated by positive EBV-DNA or EBV-IgM testing. CMV was detected in one specimen (seven specific reads). However, given the lack of supporting clinical symptoms and the relatively low read counts, infection with these viruses was not considered clinically significant.

This study represents the largest case series to date in which laboratory confirmation of CSD was achieved through mNGS detection. We anticipate that these findings may provide valuable insights for the clinical diagnosis of CSD.

However, this study had several limitations. First, its retrospective and single-center nature inherently introduced the potential for selection bias. Second, despite being one of the largest cohorts of pediatric CSD cases confirmed using mNGS, the sample size of this study remained modest. Multi-center studies with more cases should be conducted in the future to enhance a comprehensive and objective understanding of CSD. At the same time, mNGS should be optimized to reduce costs and simplify processes, thereby promoting its application in diagnosing complex cases.

## Data Availability

The datasets presented in this study can be found in online repositories. The names of the repository/repositories and accession number(s) can be found at: https://ngdc.cncb.ac.cn/gsa, CRA033062.
